# The Chemical Property Position of Bedaquiline Construed by a Chemical Global Positioning System-Natural Product

**DOI:** 10.3390/molecules27030753

**Published:** 2022-01-24

**Authors:** Muaaz Mutaz Alajlani

**Affiliations:** 1 Pharmacognosy, BMC-Biomedical Center, Department of Pharmaceutical Biosciences, Faculty of Pharmacy, Uppsala University, S-75123 Uppsala, Sweden; 2 Department of Pharmaceutical Biology/Pharmacognosy, Institute of Pharmacy, University of Halle-Wittenberg, D-06120 Halle (Saale), Germany; 3 Faculty of Pharmacy, Main Campus, Qasyoun Private University, Damascus build 131, Syria; muaaz.alajlani@qpu.edu.sy

**Keywords:** bedaquiline, antituberculosis agents, ChemGPS-NP, chemical property space, screening, structural similarity, data mining

## Abstract

Bedaquiline is a novel adenosine triphosphate synthase inhibitor anti-tuberculosis drug. Bedaquiline belongs to the class of diarylquinolines, which are antituberculosis drugs that are quite different mechanistically from quinolines and flouroquinolines. The fact that relatively similar chemical drugs produce different mechanisms of action is still not widely understood. To enhance discrimination in favor of bedaquiline, a new approach using eight-score principal component analysis (PCA), provided by a ChemGPS-NP model, is proposed. PCA scores were calculated based on 35 + 1 different physicochemical properties and demonstrated clear differences when compared with other quinolines. The ChemGPS-NP model provided an exceptional 100 compounds nearest to bedaquiline from antituberculosis screening sets (with a cumulative Euclidian distance of 196.83), compared with the different 2Dsimilarity provided by Tanimoto methods (extended connective fingerprints and the Molecular ACCess System, showing 30% and 182% increases in cumulative Euclidian distance, respectively). Potentially similar compounds from publicly available antituberculosis compounds and Maybridge sets, based on bedaquiline’s eight-dimensional similarity and different filtrations, were identified too.

## 1. Introduction

Bedaquiline (trade name Sirturo, code name TMC207) was discovered at Janssen Pharmaceutica and designated as an orphan medicinal product. Bedaquiline has proved to be a potent agent against *M. tuberculosis* H37Rv with an MIC equal to 0.03 mg/mL and against a range of *M. tuberculosis* clinical isolates with an MIC up to 0.12 mg/mL. Bedaquiline has demonstrated efficacy against resistant *M. tuberculosis* strains and activity against dormant mycobacteria [1], which have been reported as having infected over two billion human beings [2]. The mechanism of action of the diarylquinoline class of drugs was anticipated by comparing the genomic sequences of mutant resistant strains of *M. tuberculosis* and *M. smegmatis* to those that were susceptible to bedaquiline. The mutation identified at the *atpE* gene encoding for the c subunit of the ATP synthase was selective to bacteria. The compound did not inhibit gyrase activity when tested against the purified enzyme, indicating that this molecule does not share mechanism of action with quinolones [3].

Bedaquiline was successfully synthesized from 3-phenylpropionic acid and para-bromoaniline [4] and was recently approved for treating tuberculosis in standard therapy [5]. The proven superiority of bedaquiline in treating tuberculosis should not be ignored, especially as it is the first drug discovered in five decades with very encouraging clinical properties. The combination of these clinical properties has made bedaquiline an attractive medicine for the next generation of antituberculosis drugs.

In this paper, the unique and favorable properties of bedaquiline were considered by exploring its physicochemical space. This approach maintained these important physicochemical properties while profiling a similarity vector with biologically relevant compounds [6]. Such an approach has been widely overlooked due to the perceived chemical similarities that are involved.

Accordingly, the ChemGPS-NP model was used determine the number of potential candidates for bedaquiline compounds, which proved to be a far better method than focusing on chemical similarity alone. The latter approach is limited in selecting similar compounds. ChemGPS-NP demonstrated superiority in visualizing and selecting similar potential compounds in a process known as virtual screening. Finally, to maximize the efficiency of selection, selected compounds were combined for permeability profiling to further polish the outcome. In this article, an example of, and a possible raw model for, applying different techniques in antituberculosis research has been provided.

## 2. Results and Discussion

The concept of similarity, a central dogma in medicinal chemistry in which molecules may be grouped according to their chemical structures, biological effects, and physicochemical properties, has proven to be a very useful tool in drug discovery. The ranking of similarity is of particular interest in lead discovery and compound optimization. Many early forms of drugs were discovered that exhibited weak activities, unfavorable kinetics, or high toxicity. These drugs were subjected to extensive and tedious chemical iterations to produce their final approved versions. Almost all of these iteration schemes were more or less dependent on a trial-and-error method, with little efficiency. Unfortunately, most of these attempts led to astonishingly low success rates. An estimated 0.01% of chemically modified drugs were subjected too clinical trials.

On the other hand, stronger drugs with favorable kinetic, and lower toxicity came to a dead end, with chemical iterations that negatively influenced the assessment of product performance. Once again, the absence of a solid approach was accountable. 

Emerging computational methods and modeling have proved to be significant in expanding our knowledge in drug discovery, and various schemes of rational drug design have provided significant evidence in TB research [7]. Approaches using cheminformatic analysis methods [8], docking [9], Bayesian models, and a dual-event model have had different success rates [10].

**Figure 1:**
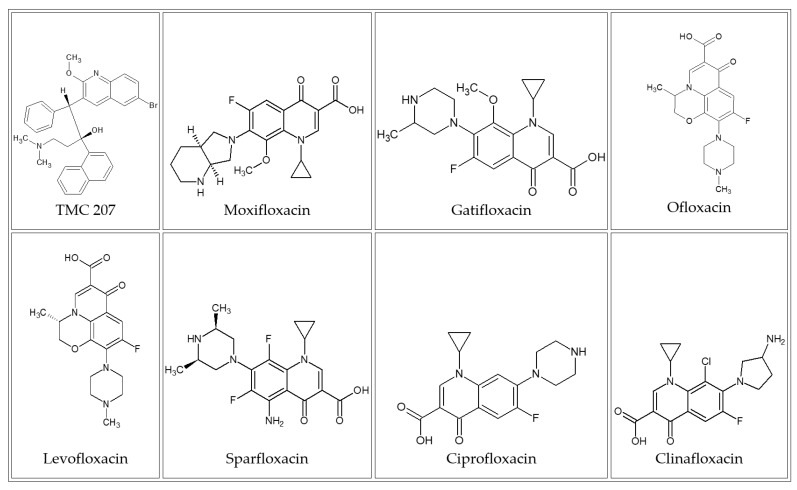
Chemical structures of bedaquiline and different typical antituberculosis quinoline compounds.

In this article, ChemGPS-NP similarity scores were used to ensure safe yet effective compound selections from huge libraries. For effective demonstration, bedaquiline was used as an example of a unique compound with distinct properties, mechanisms of action, and clinical advantages, while maintaining similarity to properties that are familiar in current medicine. Bedaquiline belongs to a class of quinoline compounds that are defined by the presence of two aromatic ring structures that are attached via a side chain to carbon 3 of the quinoline structure. Typically, the two aromatic moieties are naphthalene and benzene (Figure 1).

Quinoline-based compounds and fluoroquinolones are known to display anti-TB activities. Bedaquiline belongs to the novel chemical class known as diarylquinolines (DARQs) and displays a novel mechanism of action. Bedaquiline kills both drug-susceptible and drug-resistant *M. tuberculosis* strains, while displaying minimal inhibitory concentrations equal to or lower than standard antituberculosis medicines. It has an unusually long half-life, which is a desirable feature in support of including this drug as an intermittent regimen in a new clinical paradigm that is attracting considerable clinical interest.

Numerous compounds have been identified as possible members of this novel chemical series, while maintaining bedaquiline’s impressive anti-TB activities and possessing improved properties. The objective of this article is to propose a systematic approach for meaningful drug discovery, based on eight-dimensional space projection scores from a principle component analysis (PCA) of 35 + 1 physicochemical properties in antituberculosis compounds. These scores preserve a unique position in a large chemical property space.

**Figure 2:**
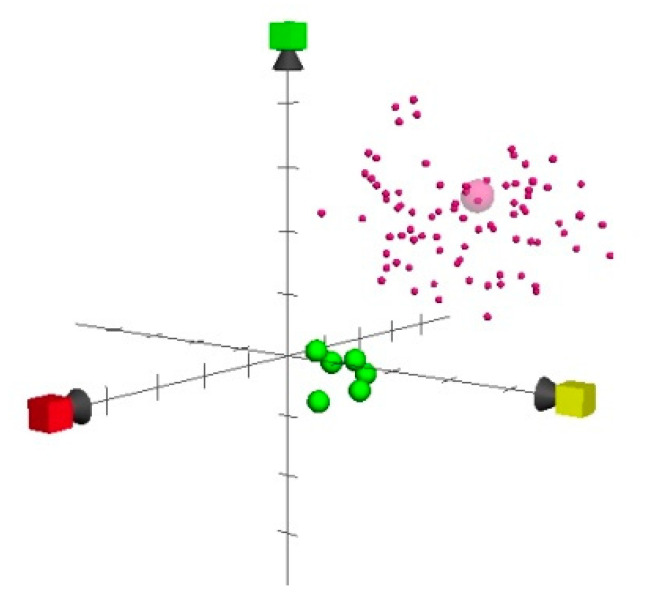
Position of bedaquiline (large pink sphere), the 100 closest antituberculosis compounds (small spheres), and quinoline compounds (green spheres), using ChemGPS-NP scores, where PC1 (x = red), PC2 (y = yellow), and PC3 (green).

Plotting compounds on such a scale makes it convenient to present the similarity and dissimilarity between different compounds. A comparative method based on the ECFP process and the MACCS keyset (MDL) was used. These approaches provide structural key-based fingerprints as an alternative to hashed fingerprints, recognizing a specific hard-coded set of chemical patterns. Only the 166-key set was implemented in this study. Fingerprints were based on structural keys from Sunset Molecular, with a current keyset size = 560. Structural keys designed for medicinal chemistry and pharmaceutical research were considered to be slightly superior for this purpose, when compared to hashed fingerprints. Their advantages over hashed fingerprints include their generality and lack of bias toward known patterns. All parameters were kept in default values. A distinction was clearly verified by plotting bedaquiline and quinolinic agents, using ChemGPS-NP scores (Figure 2).

**Figure 3:**
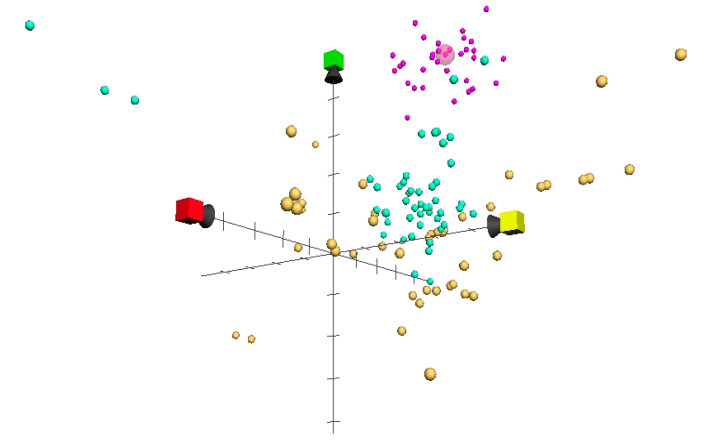
Three sets of 50 antituberculosis compounds classified as closest to bedaquiline (large pink sphere): ChemGPS-NP (pink), Tanimoto ecfp (turquoise), and Tanimoto maccos (orange), projected on a three-dimension space.

The comparative advantages of structural keys were important in mounting a novel mechanism of action in this particular agent. Moreover, it was possible to predict ATP’s novel mechanism of action idiosyncratically from the typical quinoline mechanism. The selection of compounds closest to bedaquiline was superior in maintaining its unique properties as much as possible, while avoiding the bias produced by chemical similarity modeling. As shown in Figure 3, plotting the nearest 50 compounds for each similarity modeling resulted in apparent variations between ChemGPS, ECFP, and MACCS, with accumulative eight-dimension Euclidian distant scores of 83.01425, 130.5485, and 246.0832, respectively (Supplemental Materials, S1).

These results clearly show perfect selection modeling using ChemGPS-NP scores from antituberculosis compounds, compared with chemical similarity modeling, by combining different filtration from general and antituberculosis compounds. It was possible to establish similar and potentially useful antituberculosis compounds (Supplemental Materials, S2). The similarity was achieved by calculating the shortest Euclidian distances of the eight-dimension chemical property space created by ChemGPS-NP.

**Figure 4:**
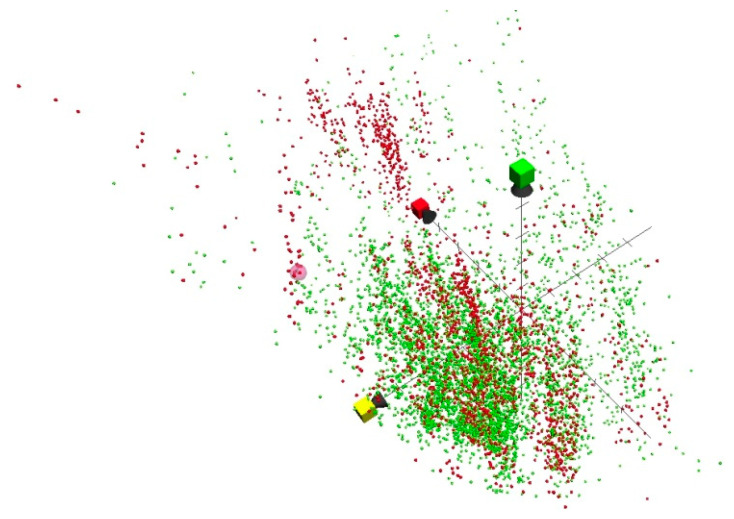
Red and green speckles represent mycobacterium permeability and impermeability, respectively. The large pink sphere represents the bedaquiline position in the chemical property space constructed by ChemGPS-NP for natural compounds.

Standard activity criteria were applied to consider antituberculosis compounds with activity of less than 5 uM. The first filtration applied, using MycPermCheck to determine permeability or impermeability of these compounds across the mycobacterial cell wall, yielded results that were presented as failed or passed. Permeability is an important factor for a successful antituberculosis agent. Projecting permeable and impermeable antituberculosis compounds in a three-dimensional chemical property space revealed the position of bedaquiline at a critical edge; hence, such filtration was of immense importance (Figure 4).

Fine-tuning of this list of compounds was carried out using SMART toxicity filtration to remove those compounds that presumably have toxicity effects (Supplemental Materials, S2). While the previous hit rate for antituberculosis compounds did not exceed 4.55% in whole-cell screening schemes, the fact that less than 0.1% ever reached clinical trial is quite disappointing.

Several limitations are often overlooked in antituberculosis compounds, including the need for a long drug half-life, reduced toxicity, permeability, effective selective activities against dormant and active mycobacteria, efficacy against resistant bacteria, co-administration with other anti-HIV drugs, fewer side effects, short treatment schemes, and low production costs. These properties can be contradictory; useful antimycobacterial compounds operate more or less between these favorable properties. The challenges presented cannot be fully satisfied by any single bioinformatics tool. ChemGPS-NP has made a positive contribution to the discovery of the next generation of antituberculosis agents.

## 3. Materials and Methods

### 3.1. Data Collection and Sources

#### 3.1.1. Basic Antituberculosis Agents

Basic drugs among the known flourquinloine class of drugs (i.e., moxifloxacin, gatifloxacin, sparfloxacin, levofloxacin, ofloxacin, and ciprofloxacin) were used to elucidate discrimination and indiscrimination between them and bedaquiline.

#### 3.1.2. Screening Sets of Antituberculosis Agents

The results of different screening schemes for antituberculosis activities, i.e., NHAID [11], GVBio5, and GSK [12], are publicly available These screening schemes were used for the efficient selection of compounds that are similar to bedaquiline.

#### 3.1.3. Drug-like Dataset

A set of compounds from the Maybridge screening collection (http://www.maybridge.com, accessed on 12 July 2014), exhibiting drug-like properties, were used as a cloud set for similarity filtration, produced by ChemGPS-NP scores.

### 3.2. Mechanism of Action

Fluoroquinolones exert their antituberculosis activities by trapping gyrase on DNA as ternary complexes, thereby blocking the movement of replication forks and transcription complexes [13]. Bedaquinline inhibits ATP synthase, a critical enzyme in the synthesis of ATP for mycobacteria [14].

### 3.3. ChemGPS-NP

ChemGPS-NP [15] (http://chemgps.bmc.uu.se, accessed on 21 October 2019) is a model of a biologically relevant chemical property space that uses principal component analysis (PCA) [16] of 35 molecular descriptors of physical-chemical properties. The model is based on eight principal components (PC) that describe a unique position in the virtual chemical property space. 

Bedaquiline and quinoline compounds were mapped and coded for their known antituberculosis mechanisms. Additional antituberculosis compounds from publicly available sources were mapped for selection purposes. The principal component and PCA score predictions were calculated via the software SIMCA-P+, with the training set ChemGPS-NP. Prior to PCA determination, all data were centered and scaled to unit variance.

### 3.4. Euclidean Distances

Based on the ChemGPS-NP model, the positions of different compound principles were calculated according to an excel formula distinguishing between a compound and a core set compounds. The Euclidean distance was calculated between points P = (*p*_1_, *p*_2_,..., *p_n_*) and Q = (*q*_1_, *q*_2_,..., *q_n_*) in Euclidean n-space, as defined by the following Equation (1):

(1) $$d\left(p,q\right)=\sqrt{{\left(p_{1}-q_{1}\right)}^{2}+{\left(p_{2}-q_{2}\right)}^{2}+\ldots +{\left(p_{i}-q_{i}\right)}^{2}+\ldots +{\left(p_{n}-q_{n}\right)}^{2}}$$

### 3.5. Permeability Prediction

Compounds from different sets that obtained higher score similarities were subjected to probability prediction for permeability, provided by MycPermCheck [17].

### 3.6. Toxicity Prediction

Toxicity examination was provided via the SmartsFilter web application from the University of New Mexico, (https://datascience.unm.edu/tomcat/biocomp/smartsfilter, accessed on 23 August 2019) [18]. Pass or fail class descriptions were assigned for each compound.

### 3.7. Structural Similarity Search

Structural fingerprints based on the MACCS keyset (MDL), together with extended connectivity fingerprints (ECFPs), were used to illustrate the Tanimoto similarity between bedaquiline and antituberculosis compounds. These fingerprints recognize a specific hard-coded set of chemical patterns that are similar to path-based patterns, using a graph showing the theory-based exhaustive enumeration of subgraph patterns [19]. Structural keys designed for medicinal chemistry and pharmaceutical research were considered slightly superior for this purpose, rather than hashed fingerprints. Their advantages over hashed fingerprints include their generality and their lack of bias toward known patterns. All parameters were maintained in default values.

## Data Availability

Supplemental data are available.

## Supplementary Materials

The following supplementary materials are available online: S1, Tanimoto similarity of TMC in different methods, including ECFP, MACCS, and Sunset; S2, List of 50 closest compounds by ChemGPS-NP.

## Sample Availability

Samples of the compounds referred to in this article are available from the author.
